# Retatrutide as a Unimolecular GIP/GLP-1/Glucagon Receptor Tri-Agonist: Molecular Design, Pharmaceutical Development and Critical Interpretation of Emerging Phase 3 Evidence

**DOI:** 10.3390/pharmaceutics18101277

**Published:** 2026-10-09

**Authors:** Diana-Maria Mateescu, Ionut Dragos Capraru, Bogdan Enache, Alexandra Enache, Cris Virgiliu Precup, Camelia Liana Buhaș, Camelia-Oana Muresan

**Affiliations:** 1 Department of Infectious Diseases, Municipal Emergency Clinical Hospital Timișoara, 2A Hector Street, 300041 Timișoara, Romania; diana.mateescu@umft.ro; 2 Department of Epidemiology, “Victor Babeș” University of Medicine and Pharmacy, Eftimie Murgu Square 2, 300041 Timișoara, Romania; ionut.capraru@umft.ro; 3 Department of Cardiology, “Victor Babeș” University of Medicine and Pharmacy of Timișoara, Eftimie Murgu Square 2, 300041 Timișoara, Romania; 4 Institute of Legal Medicine Timișoara, 1A Cireșului Street, 300610 Timișoara, Romania; muresan.camelia@umft.ro; 5 Ethics and Human Identification Research Center, “Victor Babeș” University of Medicine and Pharmacy of Timișoara, Eftimie Murgu Square 2, 300041 Timișoara, Romania; 6 Discipline of Forensic Medicine, Bioethics, Deontology, and Medical Law, Department of Neuroscience, “Victor Babeș” University of Medicine and Pharmacy of Timișoara, Eftimie Murgu Square 2, 300041 Timișoara, Romania; 7 Department of Biology and Life Sciences, Faculty of Medicine, “Vasile Goldiș” Western University of Arad, 310025 Arad, Romania; precupcris@yahoo.com; 8 Department of Morphological Disciplines, Faculty of Medicine and Pharmacy, University of Oradea, 410087 Oradea, Romania; cameliabuhas@yahoo.com

**Keywords:** retatrutide, LY3437943, triple agonist, glucagon receptor, GIP, GLP-1, peptide therapeutics, lipidation, obesity, type 2 diabetes, pharmaceutical development

## Abstract

Retatrutide (LY3437943) is an investigational, once-weekly, lipidated 39-residue peptide that activates the glucose-dependent insulinotropic polypeptide, glucagon-like peptide-1, and glucagon receptors. Its molecular design combines incretin activity with glucagon receptor engagement while maintaining glycaemic efficacy. This critical narrative review examines molecular engineering, pharmacokinetics, dose escalation, pharmaceutical development, and clinical evidence available through 11 September 2026. Five phase 3 trials have reported positive primary outcomes, but only TRANSCEND-T2D-1 has a full peer-reviewed report; the obesity trials are currently available as sponsor topline disclosures. In those disclosures, efficacy-estimand mean weight reductions of 28.3% at 80 weeks (TRIUMPH-1) and 28.7% at 68 weeks (TRIUMPH-4) were reported, with a TRIUMPH-1 treatment-regimen estimate of 25.0%; these values remain provisional pending full published trial reports. The peer-reviewed TRANSCEND-T2D-1 report supports glycaemic efficacy in early type 2 diabetes. Interpretation requires attention to estimands, population differences, treatment discontinuation and selection into trial extensions. Gastrointestinal adverse events, dysaesthesia, heart-rate changes and the functional consequences of lean-mass loss require further evaluation. Pharmaceutical priorities include scalable synthesis, impurity and potency control, formulation stability, immunogenicity and reliable dose delivery. Retatrutide provides a clinically advanced example of unimolecular poly-agonism, but comparative effectiveness, long-term outcomes and regulatory review remain essential to defining its therapeutic role.

## 1. Introduction

Obesity affects more than one billion people worldwide and is now recognised as a chronic, relapsing, multisystem disease that drives type 2 diabetes (T2D), atherosclerotic cardiovascular disease, metabolic dysfunction-associated steatotic liver disease (MASLD), obstructive sleep apnoea (OSA), osteoarthritis and several cancers [1,2]. For decades, pharmacotherapy produced weight reductions of only 5–10%, well below the 25–30% achievable with bariatric surgery. Nutrient-stimulated hormone (NuSH)-based therapies have transformed this landscape: the GLP-1 receptor (GLP-1R) agonist semaglutide 2.4 mg produced a mean weight loss of approximately 15% at 68 weeks [3,4], the GIP/GLP-1 receptor co-agonist tirzepatide approximately 21% at 72 weeks [5], and semaglutide reduced major adverse cardiovascular events in people with obesity without diabetes [6].

The addition of glucagon receptor (GcgR) agonism to incretin co-agonism was demonstrated preclinically by Finan and colleagues, who showed that a rationally designed monomeric tri-agonist improved obesity and diabetes phenotypes in rodents [7]. Retatrutide (LY3437943), developed by Eli Lilly and Company, subsequently translated this approach into clinical development [8,9]. Following phase 2 results in 2023, five phase 3 trials in the TRIUMPH and TRANSCEND-T2D programmes reported positive primary outcomes between December 2025 and July 2026, all before the 11 September 2026 evidence cutoff of this review: TRIUMPH-4 in December 2025 [10], TRIUMPH-1 in May 2026 [11], TRANSCEND-T2D-1 with full results presented and published in June 2026 [12,13], and TRIUMPH-2 and TRIUMPH-3 in July 2026 [14]. The sponsor announced plans for a US regulatory submission in the first quarter of 2027; additional clinical trials remain ongoing [10,11,12,13,14].

For a pharmaceutical-science readership, the value of an updated review extends beyond the magnitude of weight loss. This article connects receptor pharmacology and half-life extension to dose escalation, manufacturing, analytical control, formulation and device development. It also examines whether the available clinical evidence supports the mechanistic and comparative claims commonly made for triple agonism. The analysis distinguishes direct retatrutide evidence from experience with related peptides and identifies the limitations of sponsor-disclosed phase 3 results.

## 2. Scope and Approach to Evidence Synthesis

This article is a critical narrative review, not a systematic review or meta-analysis. It considers evidence available through 11 September 2026 concerning retatrutide molecular design, receptor pharmacology, pharmacokinetics, clinical efficacy and safety, and pharmaceutical development. The source set comprises original discovery and clinical publications, trial-design reports, selected mechanistic and pharmaceutical-science studies, trial registrations and official sponsor or regulatory communications. Sources were identified by searching PubMed/MEDLINE, Embase and Scopus from database inception to 11 September 2026, supplemented by ClinicalTrials.gov, the sponsor’s corporate and investor websites, and the websites of the US Food and Drug Administration and the European Medicines Agency. The core search combined the terms “retatrutide” OR “LY3437943” with terms relating to triple, tri-, or unimolecular agonism, GIP, GLP-1, and glucagon receptors, pharmacokinetics, body composition, liver fat, immunogenicity, and peptide synthesis or manufacturing. No date or language limits were applied at the search stage, although all included reports were in English. The reference lists of retrieved articles and reviews were screened for further sources, and the final search was updated on 11 September 2026, which is the evidence cutoff for this review.

Records were eligible if they reported original preclinical or clinical data on retatrutide, described the design of a retatrutide trial, provided regulatory or sponsor information about its development, or supplied mechanistic, analytical, or pharmaceutical-science context required to interpret those data. Records were excluded if they concerned other agents without bearing on the interpretation of retatrutide evidence, duplicated an included report without adding data, or consisted of unverifiable commercial or consumer material; the exception is material relating to unapproved “research-use-only” products, which is discussed in Section 9.6 as a quality and pharmacovigilance problem rather than as evidence of efficacy or safety. Sources were then prioritised in a fixed hierarchy: peer-reviewed reports of retatrutide trials took precedence over all other sources; trial registrations were used for design, population, endpoint and status details and sponsor communications were used only for phase 3 results with no full journal report in the reviewed source set. Original studies were prioritised for quantitative claims. Related-peptide studies were used to explain mechanisms or development considerations when product-specific retatrutide information was unavailable; such extrapolations are identified explicitly. Multiple reports from the same trial were treated as complementary sources rather than independent studies. Trial population, dose, duration, endpoint, estimand, and publication status were considered together when interpreting results.

The synthesis is qualitative and does not claim exhaustive retrieval, duplicate independent screening, or a formal risk-of-bias score. Particular attention is given to missing data, discontinuation, selective availability of topline outcomes, subgroup and extension analyses, and the distinction between within-trial effects and indirect comparisons. These limitations preclude pooled efficacy estimates and definitive treatment rankings.

## 3. Rationale for Unimolecular Tri-Agonism

### 3.1. From Mono- to Poly-Agonism

GLP-1 receptor (GLP-1R) agonism reduces energy intake through central pathways, delays gastric emptying, and augments glucose-dependent insulin secretion [3]. Glucose-dependent insulinotropic polypeptide receptor (GIPR) agonism adds insulinotropic activity; its effects on adipose biology and central appetite pathways are context-dependent. Preclinical studies suggest that GIPR agonism can attenuate GLP-1R agonist-associated nausea and emesis, but this mechanism should not be assumed to explain tolerability in individual patients [15,16]. The efficacy of tirzepatide supports the clinical utility of incretin co-agonism, although the relative contributions of individual receptors cannot be inferred from placebo-controlled weight-loss trials alone [5,16].

### 3.2. Glucagon Receptor Agonism: Benefits and Liabilities

Glucagon stimulates hepatic glucose production, but glucagon receptor signalling also influences hepatic lipid handling, amino-acid metabolism and, particularly in rodents, energy expenditure [17,18,19]. The rationale for tri-agonism is that concurrent incretin activity may offset glucagon-associated glucose production while retaining potentially useful metabolic effects [7,20]. Clinical glycaemic improvement supports the net efficacy of the combined molecule, rather than establishing a separately quantified benefit for each receptor. Effects on circulating amino acids, lean tissue and heart rate warrant evaluation without assuming that observations with native glucagon translate directly to chronic retatrutide exposure.

## 4. Molecular Design and Structure–Activity Considerations

### 4.1. Peptide Scaffold and Non-Coded Amino Acids

**Figure 1:**
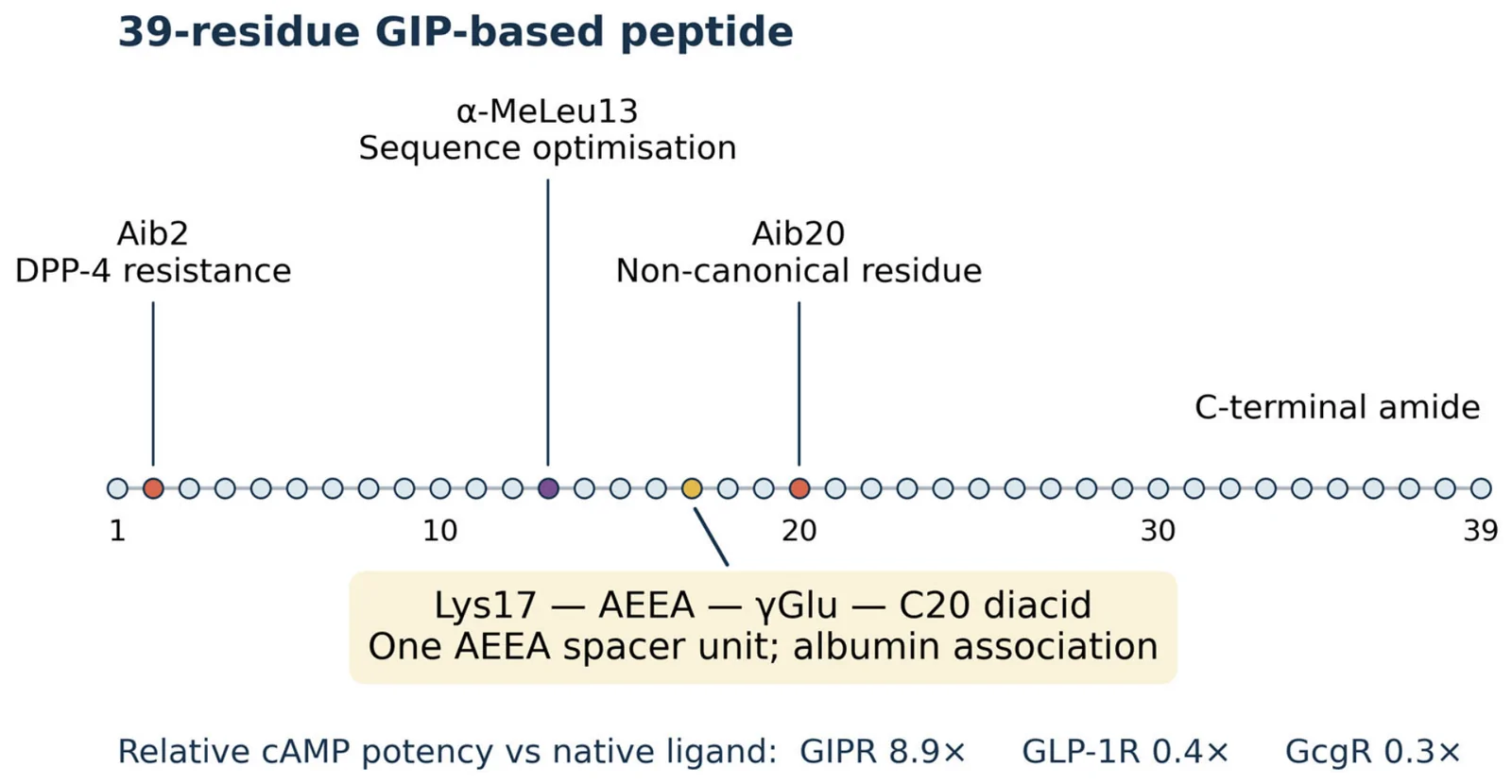
Molecular features of retatrutide. The schematic identifies the 39-residue backbone, Aib2, α-methyl-L-leucine13, Lys17 lipidation, and Aib20. The Lys17 substituent contains one AEEA unit, a γ-glutamyl group, and a C20 fatty diacid; the peptide C-terminus is amidated. The receptor potency values shown (GIPR 8.9×, GLP-1R 0.4×, GcgR 0.3×) are within-receptor comparisons: each value is expressed relative to the corresponding native ligand (GIP, GLP-1 or glucagon, respectively) at the same human receptor in cyclic adenosine monophosphate (cAMP) assays. They are not absolute potencies, should not be compared across receptors, and do not indicate tissue receptor occupancy [8]. AEEA, 8-amino-3,6-dioxaoctanoic acid; Aib, α-aminoisobutyric acid; GcgR, glucagon receptor; GIPR, glucose-dependent insulinotropic polypeptide receptor; GLP-1R, glucagon-like peptide-1 receptor.

Retatrutide is a 39-amino-acid peptide with a GIP-based sequence [8]. Its three non-proteinogenic residues are α-aminoisobutyric acid (Aib) at positions 2 and 20 and α-methyl-L-leucine at position 13; the C-terminus is amidated (Figure 1). The position-2 substitution reduces susceptibility to dipeptidyl peptidase-4 (DPP-4), while the sequence and non-canonical residues together contribute to the desired receptor-activity profile and stability [8,16,21]. Structure–activity optimisation of multi-agonists must consider the whole sequence: a substitution favourable at one receptor may alter potency at another [8,20].

### 4.2. Lipidation and Albumin-Mediated Half-Life Extension

Retatrutide is acylated at the Lys17 side chain with a C20 fatty diacid through an AEEA–γ-glutamyl spacer, where AEEA denotes 8-amino-3,6-dioxaoctanoic acid. The spacer contains one AEEA unit (Figure 1) [8]. Lipidation promotes albumin association and contributes to prolonged exposure, supporting once-weekly subcutaneous dosing. The acyl chain, linker, peptide sequence and formulation jointly influence binding and disposition; chain length alone does not establish stronger albumin binding or a longer clinical half-life than that of another analogue [8,16,21]. Amphiphilicity also makes self-association and interfacial behaviour relevant development questions, but the commercial formulation cannot be deduced from the lipidation motif.

### 4.3. Receptor Pharmacology

In human-receptor cAMP assays, retatrutide was approximately 8.9 times as potent as native GIP at GIPR and approximately 0.4 and 0.3 times as potent as native GLP-1 and glucagon at GLP-1R and GcgR, respectively [8]. These ratios derive from cAMP accumulation assays in human embryonic kidney 293 (HEK-293) clonal cell lines each expressing a single human receptor at low density (approximately 1300 GcgR, 1700 GIPR and 1400 GLP-1R receptors per cell), a design chosen to limit signal amplification and so to measure intrinsic potency. The reported half-maximal effective concentrations (EC_50_) for retatrutide were 0.0643 nM (standard error of the mean [SEM] 0.0037) at GIPR, 0.775 nM (SEM 0.041) at GLP-1R and 5.79 nM (SEM 0.28) at GcgR, against 0.574 nM, 0.312 nM and 1.97 nM for GIP(1–42)NH_2_, GLP-1(7–36)NH_2_ and glucagon, respectively; retatrutide was a full agonist at all three receptors, so the ratios reflect differences in potency rather than in maximal response, and receptor engagement was confirmed separately in radioligand binding assays [8]. Assays were run with and without human serum albumin, which is relevant because the acylated peptide binds albumin, and potencies at the corresponding mouse receptors differ from the human values, illustrating species dependence [8]. In the same report, retatrutide behaved as a partial agonist for β-arrestin recruitment at the GLP-1R, so cAMP potency does not describe its behaviour in other signalling readouts [8]. These are within-receptor comparisons against different reference ligands; they should not be read as absolute cross-receptor potency ratios or tissue-specific receptor occupancy. Apparent potency depends on receptor expression, assay conditions, species and albumin concentration. Relative cAMP potency also does not by itself establish signalling bias, receptor internalisation or long-term desensitisation [8,18]. Table 1 compares selected molecular and pharmacokinetic features with semaglutide and tirzepatide.

## 5. Integrated Pharmacology

**Figure 2:**
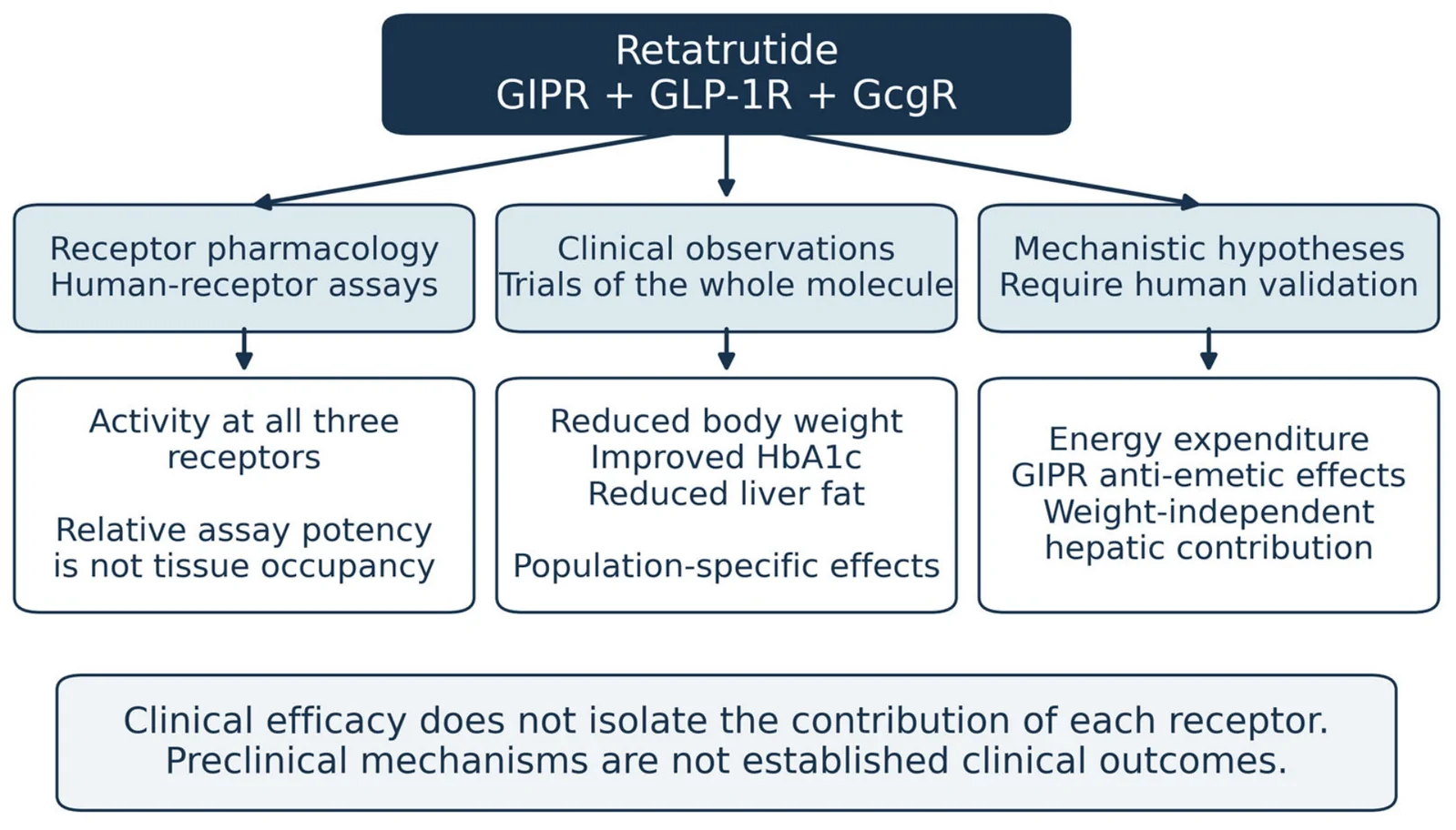
Evidence levels underlying the proposed actions of retatrutide. Receptor activity and clinical outcomes are distinguished from mechanistic extrapolation. Receptor activity refers to within-receptor cAMP potency relative to each native ligand (Figure 1; Section 4.3); these values are not absolute cross-receptor potencies and do not quantify the in vivo contribution of each receptor. Reduced weight, glycated haemoglobin (HbA1c) and liver fat are observed clinical outcomes; their relative attribution to individual receptors is unresolved. Evidence for increased energy expenditure and GIPR-mediated anti-emetic activity is principally preclinical [8,15,19,22,23].

Retatrutide integrates activity at three receptors across overlapping metabolic pathways (Figure 2). In preclinical models, reduced energy intake and increased energy expenditure both contributed to weight loss [8]. Human studies demonstrate the overall clinical effect of the molecule, but do not establish the fraction attributable to thermogenesis or to each receptor. The magnitude and persistence of any increase in human energy expenditure, therefore, remain mechanistic questions requiring direct measurement [19].

The phase 2a MASLD substudy provides direct evidence of reduced liver fat with retatrutide, rather than proof of an isolated glucagon receptor effect. Of the 338 participants in the parent obesity trial, 98 (29.1%) had liver fat ≥ 10% by magnetic resonance imaging (MRI) proton density fat fraction (MRI-PDFF) and entered the substudy: placebo (n = 19) and retatrutide 1 mg (n = 20), 4 mg (n = 19), 8 mg (n = 22) and 12 mg (n = 18). Mean baseline liver fat was approximately 19% overall, ranging from about 15.6% to 21.0% across groups. Least-squares mean relative liver-fat changes at 24 weeks were −42.9% (1 mg), −57.0% (4 mg), −81.4% (8 mg), and −82.4% (12 mg) versus +0.3% with placebo, and at 48 weeks −81.7% (8 mg) and −86.0% (12 mg) versus +4.6% with placebo. For scale, applying an approximately 81–82% relative decrease to the overall mean baseline liver-fat fraction of about 19% yields an approximate absolute reduction of 15 percentage points. This illustrative calculation is not an observed, arm-specific absolute change: baseline liver fat differed by group, and the reported least-squares mean relative changes cannot be directly converted into group-specific absolute estimates. Liver fat below 5% was reached by 27% (1 mg), 52% (4 mg), 79% (8 mg) and 86% (12 mg) of participants at 24 weeks, and by 89% and 93% of the 8 mg and 12 mg groups at 48 weeks, versus 0% with placebo [22]. Liver-fat reduction was strongly associated with weight loss and with change in visceral adipose tissue. MRI measurements were evaluable for 78.6% of substudy participants at week 24 but for only 43.9% at week 48, because of early treatment discontinuation and scans performed outside the visit window; substudy completion ranged from 58% in the placebo group to 77–90% across the retatrutide groups [22]. The week-48 values, therefore, rest on fewer than half of the randomised participants, and on a group that remained on treatment, so they carry a material risk of optimistic bias and should not be regarded as equal in reliability to the week-24 primary analysis. Group sizes of 18–22 also make the substudy imprecise, and the difference between the 8 mg and 12 mg results should not be over-interpreted. The substudy did not establish histological resolution of metabolic dysfunction-associated steatohepatitis (MASH), fibrosis improvement, or a weight-independent receptor-specific mechanism [22]. Improved glycaemia in separate diabetes trials supports the net metabolic efficacy of the combined agonist [13,23].

## 6. Clinical Pharmacology and Pharmacokinetics

### 6.1. Pharmacokinetic Profile

In the first-in-human single-ascending-dose study (0.1–6 mg subcutaneously; 45 healthy participants), exposure increased approximately in proportion to dose: geometric mean area under the concentration–time curve from time zero to infinity (AUC_0–∞_) rose from 3750 to 150,000 ng·h/mL and geometric mean maximum plasma concentration (C_max_) from 11.0 to 635 ng/mL between the 0.1 mg and 6 mg dose levels. Median time to maximum concentration (t_max_) was 12–72 h across cohorts, geometric mean terminal half-life was 134–165 h (approximately 6 days), apparent clearance was 0.027–0.043 L/h and apparent volume of distribution during the terminal phase was 5.85–8.62 L. Between-participant variability was moderate, with geometric coefficients of variation of 13–34% for AUC_0–∞_ and 10–37% for C_max_ [8]. Dose-proportional pharmacokinetics were also observed with once-weekly dosing over 12 weeks in the phase 1b study in type 2 diabetes, in which the top doses of the two highest cohorts were reached by stepwise escalation [24]. Under approximately linear kinetics and a 6-day half-life, weekly dosing would be expected to approach steady state after about four to five half-lives, that is roughly 3.5–4.5 weeks at a constant dose; because the phase 3 regimens escalate at 4-week intervals, each step lasts approximately 4.7 half-lives, so exposure is expected to approach steady state at each escalation step as well as at the maintenance dose. This is a pharmacokinetic expectation rather than a separately measured endpoint for each trial regimen: accumulation ratios, steady-state trough concentrations and a population pharmacokinetic model for retatrutide are not reported in the reviewed source set, and neither dedicated studies in renal or hepatic impairment nor a formal exposure–response analysis linking exposure to weight change, HbA1c or adverse events has been published. These gaps must be filled by product-specific characterisation before any extrapolation to prescribing recommendations.

### 6.2. Dose-Escalation Strategy to Improve Gastrointestinal Tolerability

Gastrointestinal tolerability depends on dose, escalation, and individual susceptibility. In the phase 2 obesity trial, lower starting doses reduced gastrointestinal events in groups assigned to the same maintenance dose [25]. The reported phase 3 regimens began at 2 mg weekly and increased at 4-week intervals: 2 → 4 mg; 2 → 4 → 6 → 9 mg; or 2 → 4 → 6 → 9 → 12 mg [9,11]. In TRIUMPH-1, the 4 mg regimen produced a 19.0% mean reduction in weight under the efficacy estimand and a 17.6% reduction under the treatment-regimen estimand; adverse-event discontinuation was 4.1% versus 4.9% with placebo [11]. This numerically lower discontinuation rate is not evidence of superior safety. Optimal maintenance dosing and cost-effectiveness remain to be established.

### 6.3. Drug–Drug Interaction Potential

Retatrutide-specific interaction evidence is limited, and is set out here separately from extrapolation based on related incretin therapies. Proteolytic degradation is expected to predominate over conventional cytochrome P450 (CYP)-mediated metabolism, but this expectation follows from the peptide nature of the molecule rather than from published retatrutide metabolism or interaction studies, and the absence of clinically relevant interactions requires product-specific evaluation. The one direct human observation is from a substudy of the phase 1b trial, in which retatrutide delayed gastric emptying of a liquid meal assessed by the acetaminophen absorption test: peak acetaminophen concentration fell during treatment, the effect was largest after the first dose, and it attenuated with continued dosing [26]. This demonstrates an effect on the rate of gastric emptying, but the acetaminophen test characterises liquid-phase emptying and does not establish that the exposure of any particular co-administered medicine is meaningfully altered. Delayed gastric emptying may alter the absorption rate or exposure of concomitant oral medicines, constituting a potential pharmacokinetic interaction. Oral drugs requiring reliable exposure or rapid onset deserve particular attention. The remaining considerations are extrapolations from related agents and should be read as hypotheses for testing. Reduced oral contraceptive exposure is a recognised concern with tirzepatide, for which the prescribing information advises switching to a non-oral method or adding a barrier method for 4 weeks after initiation and after each dose escalation [27], but no dedicated drug–drug interaction study of retatrutide with an oral contraceptive, or with any other narrow-therapeutic-index or rapid-onset oral medicine, has been reported in the reviewed source set; equivalent instructions, therefore, cannot be assigned to retatrutide, and any effect on oral-drug absorption requires direct confirmation before it is reflected in labelling. Separately, concomitant insulin or sulfonylureas may increase hypoglycaemia risk through a pharmacodynamic interaction. The dose-adjustment and monitoring approach must be established in the relevant clinical populations.

## 7. Clinical Efficacy

**Figure 3:**
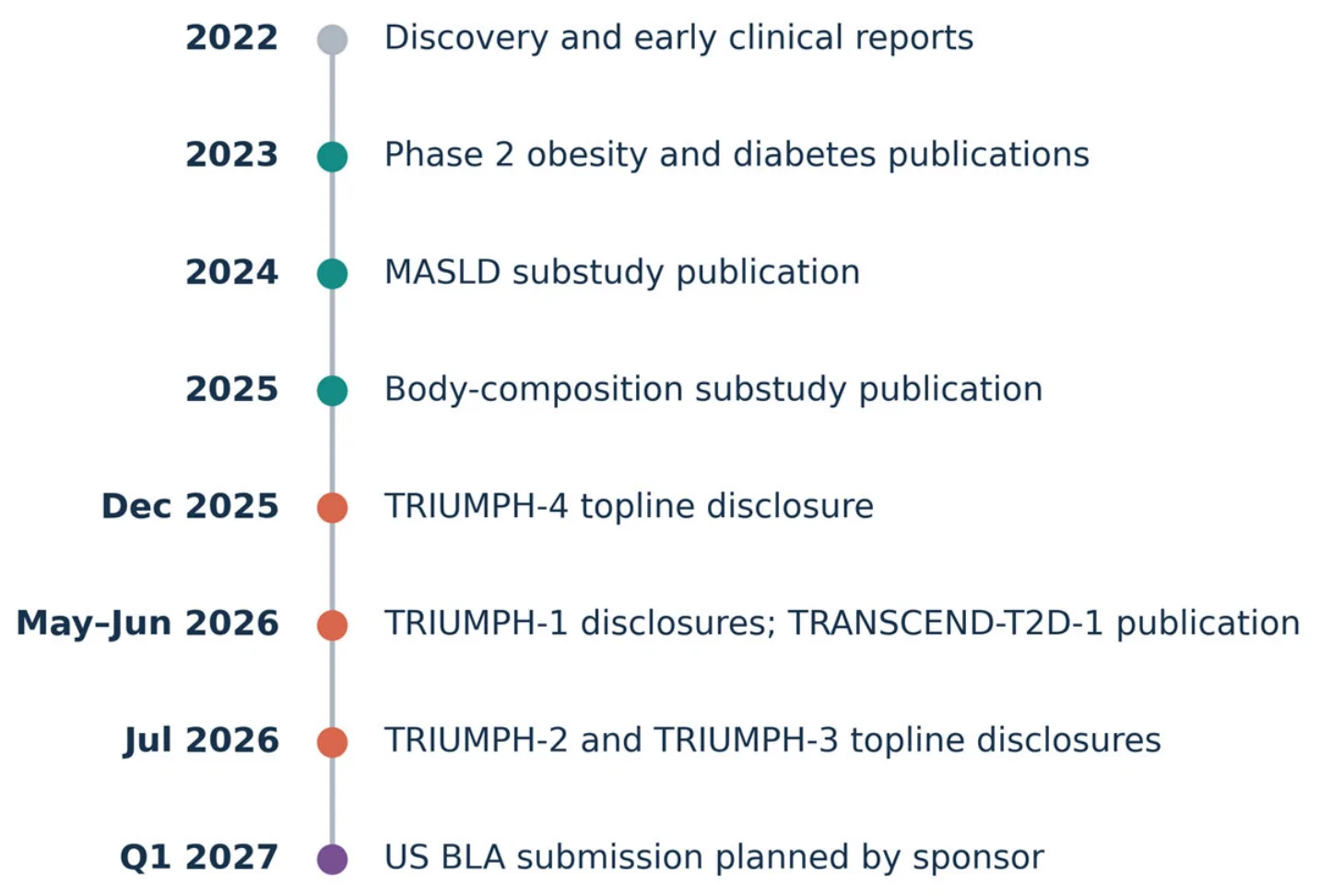
Selected retatrutide development milestones. Dates refer to publication years for discovery and phase 2 reports and to sponsor announcement dates for phase 3 results; TRANSCEND-T2D-1 is shown at the date of its full presentation and peer-reviewed publication. Colors indicate the type of developmental milestone: gray represents discovery and early clinical reports; teal represents peer-reviewed scientific publications; orange represents phase 3 sponsor-disclosed results (with the May–June 2026 milestone also including the peer-reviewed TRANSCEND-T2D-1 publication); and purple represents the sponsor’s projected regulatory submission. All milestones up to July 2026 precede the 11 September 2026 evidence cutoff. The planned US submission is a sponsor forecast, not an approval milestone. Additional trials remain ongoing [8,10,11,12,13,14,22,23,24,25,26,28].

The clinical development of retatrutide, from first-in-human studies through phase 3 readouts and the planned US regulatory submission, is summarized in Figure 3.

### 7.1. Phase 1 and Phase 2 Studies

The phase 1b study in T2D demonstrated dose-dependent reductions in HbA1c and body weight over 12 weeks and established the need for gradual escalation to mitigate gastrointestinal events [24]. Two phase 2 trials, both published in 2023, then established clinical proof of concept (Table 2). In adults with obesity without diabetes (n = 338), mean weight reductions at 48 weeks were 8.7%, 17.1%, 22.8% and 24.2% with 1, 4, 8 and 12 mg, respectively, versus 2.1% with placebo, and the weight trajectory had not plateaued at the highest dose [25]. In T2D (n = 281), retatrutide reduced HbA1c by up to 2.02 percentage points at 24 weeks—exceeding the reduction with dulaglutide 1.5 mg—and body weight by up to 16.9% at 36 weeks [23]. The MASLD substudy of the obesity trial, described above, provided the first clinical evidence of pronounced hepatic effects [22].

### 7.2. The Phase 3 Programmes: TRIUMPH and TRANSCEND-T2D

**Figure 4:**
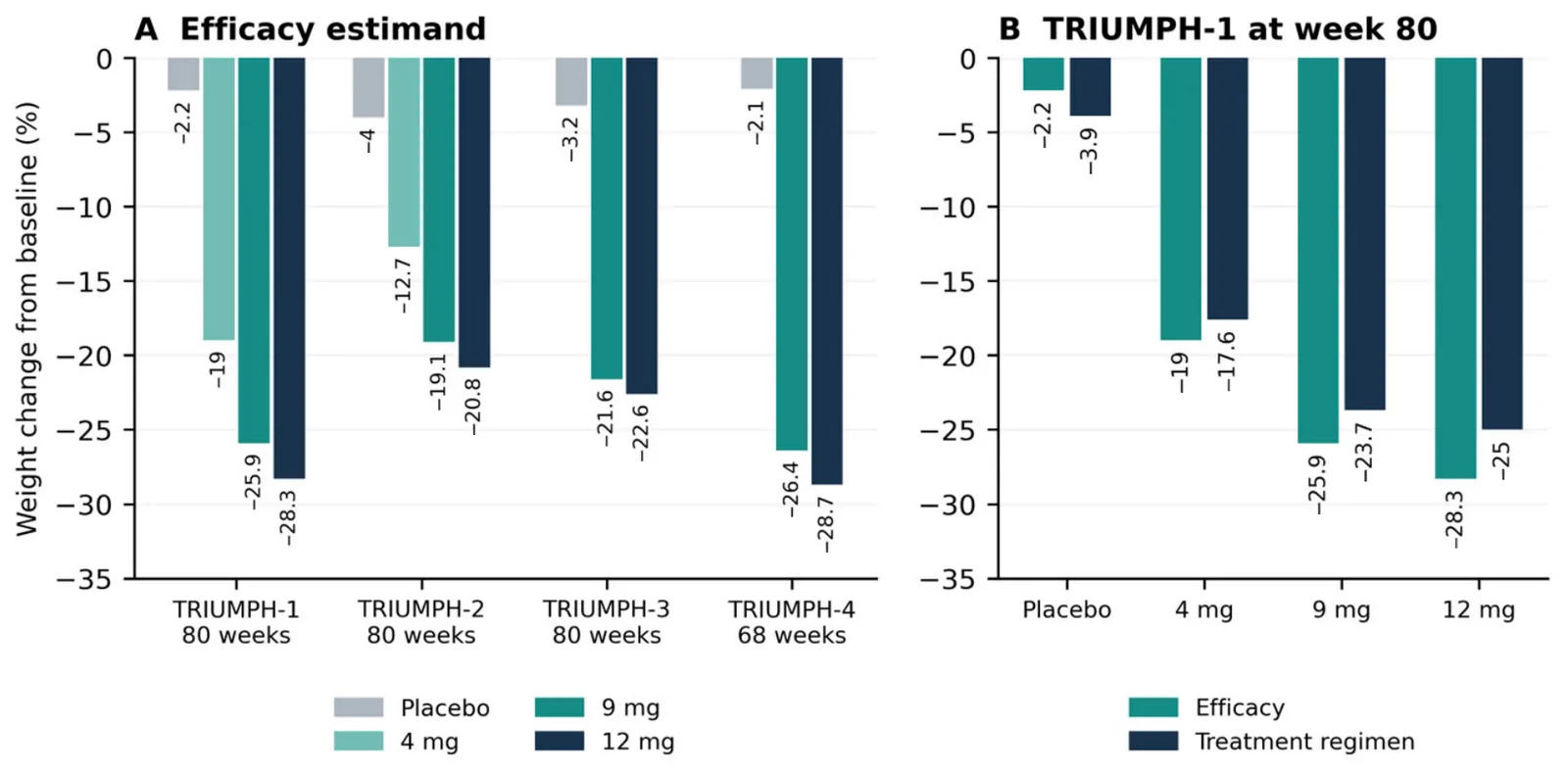
Reported weight outcomes in the TRIUMPH trials. (**A**) Efficacy-estimand mean percentage changes in body weight at each trial’s primary assessment. (**B**) Efficacy-estimand and treatment-regimen estimates in TRIUMPH-1 at week 80. Both panels use within-group changes from baseline. All values are from sponsor topline disclosures and are not yet supported by full peer-reviewed reports. Trials differ in population and duration; the graphic is descriptive and does not support head-to-head inference. Confidence intervals were not supplied for these values in the cited topline tables and are, therefore, not plotted [10,11,14].

The initial TRIUMPH programme enrolled more than 5800 participants across four registrational trials, including an overarching obesity trial with nested condition-specific baskets [9]. The TRANSCEND-T2D programme evaluates glycaemic efficacy in diabetes; the monotherapy trial has a peer-reviewed report [13]. Table 3 separates trial populations and states the evidence status of each trial explicitly: at the evidence cutoff, TRANSCEND-T2D-1 is the only phase 3 trial with a full peer-reviewed report [13], whereas the weight, comorbidity and safety results of TRIUMPH-1 to TRIUMPH-4 derive from sponsor topline disclosures and congress presentations [10,11,12,14]. Topline disclosures typically report selected endpoints without the full analysis populations, confidence intervals, sensitivity analyses or missing-data accounting that a complete report provides, so all TRIUMPH values quoted below should be read as provisional. Figure 4 presents weight outcomes descriptively, without ranking treatments across different trials.

TRIUMPH-1 randomised 2339 adults without diabetes to retatrutide 4, 9 or 12 mg or placebo for 80 weeks. Mean baseline body mass index (BMI) was 40.0 kg/m^2^. Efficacy-estimand weight changes were −19.0%, −25.9% and −28.3%, respectively, versus −2.2%; treatment-regimen changes were −17.6%, −23.7% and −25.0% versus −3.9% [11]. At 12 mg, 45.3% achieved ≥30% weight loss and 27.2% achieved ≥35%. The nested osteoarthritis and OSA trials reported improvements in pain and apnoea–hypopnoea index (AHI), respectively [12]. These comorbidity findings should be interpreted with their own populations, baseline severity, comparators and endpoints, rather than as outcomes in every participant in the overarching trial [9,11,12]. Published tirzepatide trials in OSA provide related clinical context, but do not permit a direct comparison with retatrutide [29].

The 104-week extension included 532 participants with baseline BMI ≥ 35 kg/m^2^ who completed the 80-week period and tolerated assigned treatment. All extension groups received retatrutide at a maximum tolerated dose of 9 or 12 mg, including the original placebo group. Weight reduction reached 30.3% in the group originally assigned 12 mg under the efficacy estimand [11]. This selected, treatment-tolerant extension population and the change in comparator treatment prevent direct interpretation as an unselected, placebo-controlled 104-week effect.

TRIUMPH-4 enrolled 445 adults with obesity or overweight and knee osteoarthritis. At 68 weeks, sponsor-reported efficacy-estimand weight changes were −26.4% with 9 mg and −28.7% with 12 mg versus −2.1% with placebo. Treatment-regimen weight changes were −20.0% and −23.7% versus −4.6%, respectively. On the efficacy estimand, Western Ontario and McMaster Universities Osteoarthritis Index (WOMAC) pain scores (0–10 scale) changed by −4.5 and −4.4 points versus −2.4 with placebo [10]. The weight and symptom findings are clinically relevant, but do not establish structural joint modification. Comparisons with semaglutide in STEP 9 remain indirect because the populations, interventions, and follow-up differ [30].

TRIUMPH-2 randomised 1152 participants with type 2 diabetes and obesity or overweight. At 80 weeks, efficacy-estimand weight changes were −12.7%, −19.1% and −20.8% with 4, 9 and 12 mg, respectively, versus −4.0% with placebo; HbA1c changes were −1.4, −1.6 and −1.5 percentage points versus −0.2 [14]. Differences from non-diabetes trials may reflect several population and treatment factors. SURMOUNT-2 supplies useful context for tirzepatide in diabetes, but its results do not permit a causal comparison with retatrutide [31].

TRIUMPH-3 randomised 1949 adults with BMI ≥ 35 kg/m^2^ and established cardiovascular disease, with or without diabetes, to 9 mg, 12 mg or placebo in a 1:1:2 ratio. At 80 weeks, efficacy-estimand weight changes were −21.6%, −22.6% and −3.2%, respectively [14]. In the prespecified in-study analyses comparing pooled retatrutide doses with placebo, the MACE-5 (all-cause death, myocardial infarction, stroke, heart-failure event or coronary revascularisation) hazard ratio was 0.82 (95% confidence interval [CI] 0.55–1.22; 44 versus 52 events), and the MACE-3 (cardiovascular death, myocardial infarction or stroke) hazard ratio was 1.12 (0.64–1.96; 27 versus 23 events). Neither estimate establishes cardiovascular benefit or excludes clinically relevant harm. The dedicated TRIUMPH-Outcomes trial is needed to clarify cardiovascular and renal outcomes [32].

**Table 3:** Principal results of phase 3 trials of retatrutide reported as of 11 September 2026 (evidence cutoff).

Trial/Registration	Population; *n*	Duration	Weight Change (%)	Other Outcomes	Evidence Status
TRIUMPH-1 [33]	Obesity/overweight without T2D; 2339	80 weeks	Efficacy: 4/9/12 mg −19.0/−25.9/−28.3; PBO −2.2. Regimen: −17.6/−23.7/−25.0; PBO −3.9.	Nested OA and OSA trials reported symptom improvements.	Sponsor topline disclosure; congress presentation; no full report [11,12]
TRIUMPH-2 [34]	T2D and obesity/overweight; 1152	80 weeks	Efficacy: 4/9/12 mg −12.7/−19.1/−20.8; PBO −4.0.	HbA1c: −1.4/−1.6/−1.5 pp; PBO −0.2.	Sponsor topline disclosure; no full report [14]
TRIUMPH-3 [35]	BMI ≥ 35 kg/m^2^; established CVD ± T2D; 1949	80 weeks	Efficacy: 9/12 mg −21.6/−22.6; PBO −3.2.	MACE analyses remain inconclusive; see text.	Sponsor topline disclosure; no full report [14]
TRIUMPH-4 [36]	Obesity/overweight and knee OA; 445	68 weeks	Efficacy: 9/12 mg −26.4/−28.7; PBO −2.1. Regimen: −20.0/−23.7; PBO −4.6.	WOMAC pain: −4.5/−4.4 vs. −2.4 points (efficacy).	Sponsor topline disclosure; no full report [10]
TRANSCEND-T2D-1 [37]	T2D on diet/exercise alone; 537	40 weeks	Regimen: 4/9/12 mg −11.5/−13.9/−15.3; PBO −2.6.	HbA1c: −1.69/−1.86/−1.94 pp; PBO −0.81.	Peer-reviewed full report [13]

Data from refs. [10,11,12,13,14,33,34,35,36,37]. Weight changes are within-group changes from baseline, not placebo-adjusted effects. Efficacy and treatment-regimen estimates are identified separately. Extension participants were selected completers who tolerated treatment and received maximum tolerated retatrutide. BMI, body mass index; CVD, cardiovascular disease; MACE, major adverse cardiovascular events; OA, osteoarthritis; OSA, obstructive sleep apnoea; PBO, placebo; pp, percentage points; T2D, type 2 diabetes; WOMAC, Western Ontario and McMaster Universities Osteoarthritis Index.

TRANSCEND-T2D-1 randomised 537 adults with type 2 diabetes inadequately controlled by diet and exercise alone; mean baseline HbA1c was 7.9% [13]. At 40 weeks, treatment-regimen HbA1c changes were −1.69, −1.86 and −1.94 percentage points with 4, 9 and 12 mg versus −0.81 with placebo. Weight changes were −11.5%, −13.9% and −15.3% versus −2.6%. The sponsor additionally reported an efficacy-estimand weight change of −16.8% with 12 mg [12,13]. This distinction matters because the estimates answer different treatment questions. The population does not establish effectiveness in insulin-treated or more advanced diabetes.

### 7.3. Interpreting Estimands and Cross-Trial Comparisons

The International Council for Harmonisation (ICH) E9(R1) framework requires specification of the population, treatment conditions, endpoint, handling of intercurrent events, and population-level summary [38]. In broad terms, the efficacy estimand targets the hypothetical effect under continued assigned treatment without specified additional therapies, while the treatment-regimen estimand targets the effect of assignment regardless of subsequent discontinuation or additional therapy; the precise questions depend on each trial’s protocol. The labels describe different strategies for handling treatment discontinuation and additional or prohibited therapies, but they do not specify the statistical method for incomplete outcome data; trial-specific definitions and reported missing-data methods are compared in Table 4 [10,11,13,14]. Differences between the estimates reflect the handling of intercurrent events and missing data, not a separately identified causal effect of discontinuation. The 3.3-percentage-point difference for 12 mg in TRIUMPH-1 should, therefore, be reported without assigning a single explanation. A direct randomised comparison with tirzepatide in TRIUMPH-5 should reduce between-trial confounding, although its interpretation will still depend on trial conduct, missingness and estimands [39].

## 8. Safety and Tolerability

**Figure 5:**
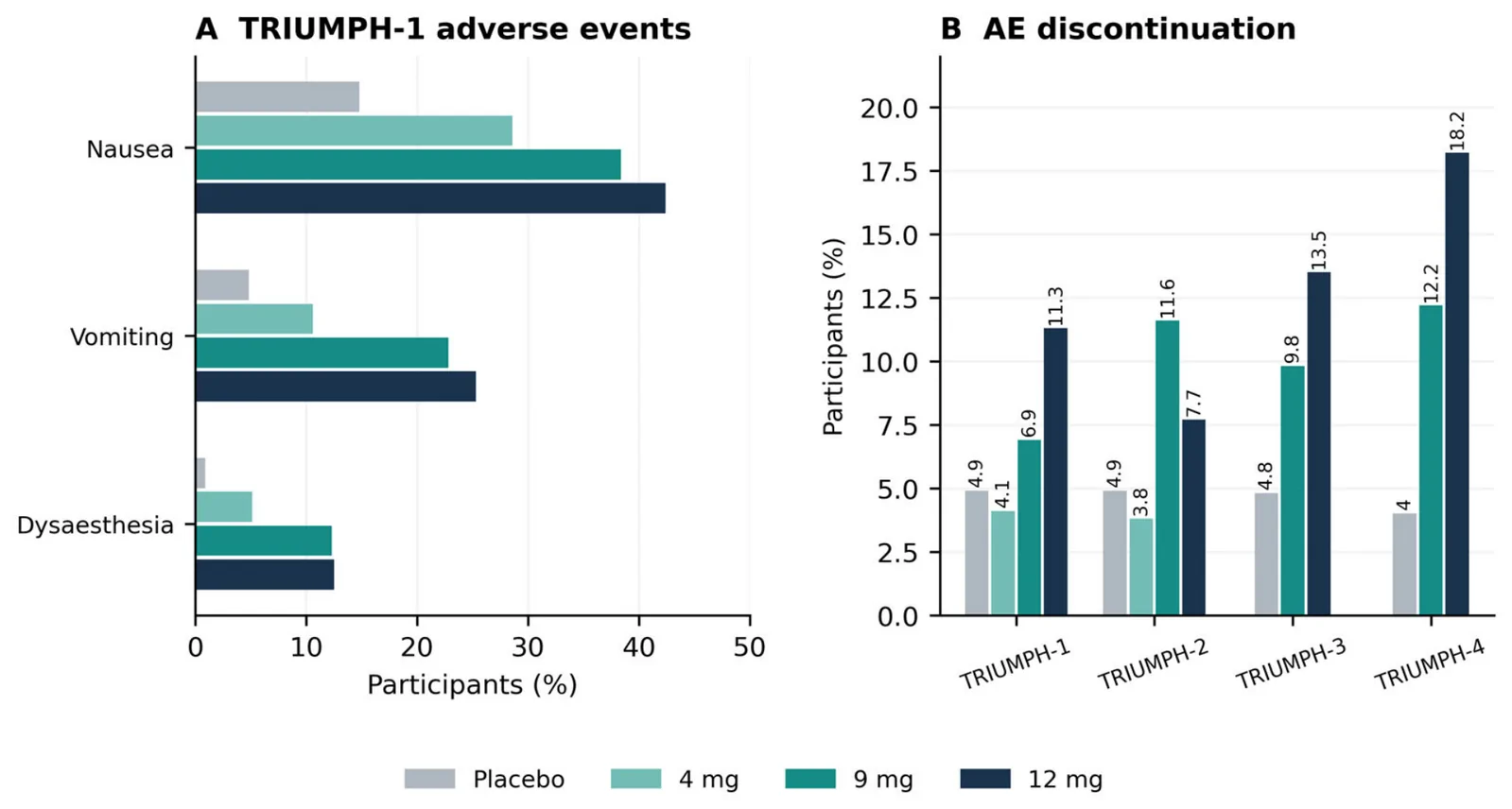
Selected adverse events and discontinuation. (**A**) Reported frequencies of nausea, vomiting and dysaesthesia in TRIUMPH-1. (**B**) Discontinuation due to adverse events in TRIUMPH-1 to -4. All values are from sponsor topline disclosures, not peer-reviewed reports. Frequencies are descriptive; differences in trial populations, follow-up and treatment exposure preclude direct safety rankings [10,11,14].

The safety profile of retatrutide is broadly consistent with the NuSH agonist class but displays several distinguishing features (Table 5, Figure 5).

Gastrointestinal events. Nausea, diarrhoea, constipation and vomiting were frequent and tended to occur during escalation [11,14]. In TRIUMPH-1, nausea occurred in 28.6%, 38.4% and 42.4% of participants receiving 4, 9 and 12 mg versus 14.8% with placebo; corresponding vomiting frequencies were 10.6%, 22.8% and 25.3% versus 4.8% [11]. Lower frequencies for some events in other trials should not be interpreted as better tolerability in a particular population without accounting for exposure, ascertainment and discontinuation. In TRANSCEND-T2D-1, adverse-event discontinuation was 2.2%, 4.5% and 5.1% with 4, 9 and 12 mg versus 0% with placebo [12,13].

Dysaesthesia. Abnormal skin sensations were reported in 5.1–12.5% of retatrutide groups versus 0.9% with placebo in TRIUMPH-1, and in 20.9% of the 12 mg group in TRIUMPH-4 [10,11]. This warrants targeted assessment, even though the sponsor described most events as mild or moderate and many as resolving during treatment. The available data do not establish a mechanism, an exposure threshold, or reversibility in all affected participants. A causal explanation involving small-fibre injury or direct sensory-neuron signalling would require dedicated evidence.

Heart rate and cardiovascular safety. The phase 2 obesity study reported dose-dependent increases in heart rate that peaked at approximately week 24 and subsequently declined [25]. Attribution specifically to glucagon receptor agonism is uncertain. Integrated evaluation should include resting heart rate, arrhythmias, symptoms, treatment exposure, and adjudicated cardiovascular events. Improvements in blood pressure or lipids cannot substitute for cardiovascular outcome evidence, particularly in light of the imprecise TRIUMPH-3 estimates [14].

Urinary tract infections and treatment discontinuation. Urinary tract infections were reported in 7.5–8.8% of retatrutide groups versus 5.3% with placebo in TRIUMPH-1; causality and mechanism are not established [11]. Adverse-event discontinuation reached 18.2% with 12 mg in TRIUMPH-4, and the sponsor described greater discontinuation among participants with lower baseline BMI, including perceived excessive weight loss [10]. Rates were not monotonically dose-related in every trial: in TRIUMPH-2, they were 3.8%, 11.6% and 7.7% with 4, 9 and 12 mg [14]. These observations support investigation of individualised maintenance strategies, not an established dose-selection algorithm.

Unresolved safety questions. Pancreatitis, gallbladder events, severe gastrointestinal intolerance, aspiration during anaesthesia, retinopathy changes accompanying rapid glycaemic improvement and thyroid C-cell findings are areas for assessment in the integrated safety programme. Risks associated with approved related agents should not automatically be treated as demonstrated retatrutide risks. Rare events, pregnancy exposure and long-term effects require larger datasets and dedicated follow-up.

Body composition. A published phase 2 dual-energy X-ray absorptiometry (DXA) substudy in adults with type 2 diabetes assessed the effects of retatrutide on fat and lean mass [28]. Thus, body-composition evidence is not entirely absent. These findings do not establish long-term preservation of muscle strength, physical performance, or bone health, particularly in older adults or people with sarcopenia. Lean mass measured by DXA is not equivalent to skeletal muscle mass or function. Future trials should pair body-composition measurements with functional and nutritional assessments rather than treating total weight loss as a sufficient measure of benefit.

## 9. Pharmaceutical Development, Quality and Regulatory Considerations

The available reports do not disclose a complete commercial chemistry, manufacturing and controls (CMC) package. The sponsor stated that this package was being completed before its planned regulatory submission [14]. Accordingly, Table 6 separates known molecular features from development questions. The discussion below uses published experience with related peptides to identify plausible considerations and does not purport to describe Lilly’s undisclosed manufacturing process or final formulation.

### 9.1. Drug-Substance Manufacturing

The non-proteinogenic residues and site-specific lipidation complicate straightforward recombinant production and make chemical synthesis a plausible approach. A hybrid solid-phase/liquid-phase peptide synthesis (SPPS/LPPS) process has been described for tirzepatide, including kilogram-scale good manufacturing practice (GMP) manufacture [40]. It provides a relevant manufacturing precedent but is not evidence of the route used for retatrutide. Potential challenges include incomplete coupling, sterically hindered residues, regioselective acylation, epimerisation at susceptible stereocenters, purification burden and solvent use. Process yield, impurity clearance and batch consistency need to be demonstrated rather than inferred from structural similarity.

### 9.2. Critical Quality Attributes and Impurity Control

A structure-informed control strategy, organised around critical quality attributes (CQAs), would consider sequence-related impurities, stereochemical variants at chiral residues, incomplete or incorrect acylation, relevant degradation products and aggregates. The relevance of each impurity must be established experimentally; the mere presence of a peptide bond does not imply that every common peptide degradation pathway is equally important. US Food and Drug Administration (FDA) guidance on selected highly purified synthetic peptides provides a useful precedent for impurity characterisation and immunogenicity assessment, but concerns specific abbreviated new drug applications and is not a retatrutide-specific regulatory standard [41].

Orthogonal methods can address different quality attributes: chromatographic separation and mass spectrometry for sequence and acylation identity; suitable assays for peptide content and related substances and particle or aggregation measurements appropriate to the product. For a tri-agonist, chemical purity alone cannot demonstrate the intended biological activity at all three receptors. Receptor-specific functional assays, qualified reference material and an appropriate potency-control strategy would connect manufacturing consistency with pharmacological performance. Sterility, bacterial endotoxins, and container-closure integrity additionally apply to the finished injectable product [8,41].

### 9.3. Formulation, Stability and Device

Formulation development must accommodate the amphiphilic peptide while maintaining chemical stability, acceptable particle levels and reproducible delivery. Candidate variables include pH, ionic strength, peptide concentration, tonicity agents and, for multidose presentations, preservative compatibility. Stability studies should evaluate the selected formulation under relevant storage, transport, and in-use conditions, including contact with container and device materials. These are development considerations, not measured retatrutide formulation characteristics. The clinical escalation doses of 2, 4, 6, 9 and 12 mg do not establish the number of commercial presentations: device configuration, delivered volume, dose accuracy and human-factors validation must be considered together. A multidose design could alter supply requirements but introduces its own in-use stability and dosing risks.

### 9.4. Immunogenicity

Immunogenicity assessment should distinguish treatment-emergent binding anti-drug antibodies (ADAs) from neutralising activity, titre, persistence and effects on exposure, efficacy or hypersensitivity. Potential cross-reactivity with native GIP, GLP-1 or glucagon is a question for validated assays rather than an established clinical phenomenon. Neutralisation of endogenous glucagon would be relevant to counter-regulation, but cannot be inferred from antibody positivity alone. Integrated retatrutide-specific results, including assay sensitivity and drug tolerance, are needed before drawing conclusions about the clinical significance of antibody responses [8,13,41].

### 9.5. Regulatory Pathway

In July 2026, Lilly announced plans to submit a Biologics License Application (BLA) in the United States in the first quarter of 2027 after completing its CMC package [14]. This is a sponsor-stated plan and should not be represented as regulatory acceptance of a filing, an approval date, or a final classification decision. Regulatory requirements depend on jurisdiction and product-specific determinations; a comparison of peptide length alone cannot resolve the applicable pathway. Retatrutide remains investigational, and its eventual indications, contraindications, presentation, and prescribing instructions require regulatory review [14,42].

### 9.6. Unregulated ‘Research-Use-Only’ Retatrutide: A Quality and Pharmacovigilance Challenge

FDA identifies retatrutide as an unapproved substance and states that it cannot be used in compounding under US federal law. The agency has also warned about products marketed to consumers while labelled for research purposes or not for human consumption [42]. Product quality, identity, and dose cannot be assumed from an online vendor’s description or an isolated high-performance liquid chromatography (HPLC) chromatogram. A chromatographic area percentage is not equivalent to peptide content, receptor potency, sterility, or freedom from endotoxin. Unverified reconstitution and administration add opportunities for contamination and dosing error. Suspected adverse events from these products cannot reliably quantify the intrinsic safety profile of trial-grade retatrutide because identity, exposure, and co-use may be unknown. Analytical investigation and pharmacovigilance should document the product source and quality uncertainty, rather than treating online self-reports as a substitute for controlled clinical evidence.

## 10. Positioning Within the Next-Generation Obesity Pharmacotherapy Landscape

Retatrutide is being developed alongside higher-dose GLP-1R agonists, amylin-containing combinations, oral small-molecule agonists and other glucagon-containing agents [43,44,45,46]. Table 7 lists selected trial benchmarks for context only. These are not comparative-effectiveness estimates, and the trials differ in population, baseline body mass index, duration, dose and escalation schedule, background care, estimand definition and missing-data handling, any one of which can shift a within-group percentage change by several points. Indirect comparison across these trials is, therefore, not a valid basis for ranking agents, and no such ranking is offered here; at the evidence cutoff, no completed head-to-head trial of retatrutide against semaglutide, tirzepatide or any other agent has been reported, and TRIUMPH-5 (versus tirzepatide) and TRANSCEND-T2D-2 (versus semaglutide in type 2 diabetes) are the randomised comparisons that could eventually support such statements [39,47]. The large weight reductions reported in TRIUMPH make retatrutide relevant to the management of obesity and its complications, but evidence does not yet define preferential use by BMI category, prior treatment response, or cost. Survodutide has also progressed to phase 3: SYNCHRONIZE-MASLD evaluated adults with obesity and at-risk MASLD [48]. Such studies inform the broader development landscape but cannot isolate the benefit of adding GIPR activity because the compounds differ in receptor pharmacology, dose, and trial populations.

## 11. Knowledge Gaps and Future Directions

Several questions remain central to retatrutide’s clinical value. TRIUMPH-Outcomes must clarify cardiovascular and renal endpoints; TRIUMPH-3 does not establish cardiovascular protection [14,32]. Body-composition work should extend the published phase 2 evidence to physical function, strength, nutrition and bone health across broader populations [28]. Maintenance and withdrawal studies are needed to examine durability, weight regain and the consequences of dose reduction. Dysaesthesia and heart-rate changes require exposure-informed follow-up. Direct calorimetry and mechanistic studies should test the proposed contribution of glucagon receptor signalling in humans. Evidence in older, frail and organ-impaired populations should guide, rather than follow, broad clinical extrapolation.

For pharmaceutical development, priorities are a transparent, validated control strategy linking molecular identity and three-receptor potency; scalable manufacture with documented impurity clearance; stability in the selected container and device and immunogenicity assays with demonstrated interpretability. Exposure–response analyses may help evaluate titration and maintenance strategies. Longer-acting or oral delivery approaches remain research proposals and would require their own absorption, stability, and safety evidence. Surveillance of unapproved products is a separate public-health priority and must not blur the distinction between trial material and products of uncertain provenance.

## 12. Conclusions

Retatrutide demonstrates that a single engineered peptide can combine GIPR, GLP-1R and glucagon receptor activity with a pharmacokinetic profile compatible with weekly administration. Phase 2 publications and emerging, largely sponsor-reported, phase 3 results support substantial effects on weight and glycaemia, with additional findings for liver fat and obesity-related symptoms. Interpretation remains conditional on evidence provenance, estimand choice, tolerability and population. Consistent with the narrative design of this review, no pooled efficacy or safety estimates or treatment rankings are provided; the cross-trial tables and figures are descriptive benchmarks, not comparative-effectiveness evidence. Neither the relative contribution of each receptor nor superiority to other treatments is established by these placebo-controlled studies. Pharmaceutical development must connect molecular design to reproducible potency, product quality, stability and dose delivery. Full clinical reports, integrated safety and immunogenicity analyses, direct comparisons and outcome trials will determine whether the reported efficacy translates into a favourable and durable benefit–risk profile.

## Figures and Tables

**Table 1:** Selected molecular and pharmacokinetic characteristics of semaglutide, tirzepatide and retatrutide.

Characteristic	Semaglutide	Tirzepatide	Retatrutide
Receptor targets	GLP-1R	GIPR; GLP-1R	GIPR; GLP-1R; GcgR
Peptide backbone	GLP-1 analogue; 31 aa	GIP-based; 39 aa	GIP-based; 39 aa
Non-canonical residues	Aib8 (native numbering)	Aib2; Aib13	Aib2; α-MeLeu13; Aib20
Lipidation site	Lys26	Lys20	Lys17
Acyl substituent	C18 diacid with γGlu and two AEEA units	C20 diacid with γGlu and two AEEA units	C20 diacid with γGlu and one AEEA unit
Approximate elimination half-life, t½ (days)	7	5	6
Selected weekly s.c. trial doses (mg)	2.4	5, 10, 15	4, 9, 12 (investigational)

Data from refs. [8,16,21]. Doses are selected once-weekly subcutaneous regimens discussed in this review, not a complete statement of current product labelling. Semaglutide residue positions follow native GLP-1 numbering; tirzepatide and retatrutide positions follow their 39-residue sequences. aa, amino acids; AEEA, 8-amino-3,6-dioxaoctanoic acid; Aib, α-aminoisobutyric acid; GcgR, glucagon receptor; GIPR, glucose-dependent insulinotropic polypeptide receptor; GLP-1R, glucagon-like peptide-1 receptor; s.c., subcutaneous; t½, elimination half-life; α-MeLeu, α-methyl-L-leucine; γGlu, γ-glutamyl.

**Table 2:** Summary of phase 1 and phase 2 clinical studies of retatrutide (peer-reviewed).

Study	Population/Design	Duration	Key Efficacy Findings	Key Tolerability Findings
Phase 1 SAD [8]	Healthy participants; single ascending doses	Single dose	t½ ≈ 6 days; dose-dependent PK	GI events dose-related
Phase 1b MAD [24]	T2D; multiple ascending doses vs. placebo	12 weeks	Dose-dependent ↓HbA1c and ↓body weight	Escalation required to limit GI events; ↑heart rate
Phase 2 obesity [25]	Obesity/overweight without T2D; n = 338; 1–12 mg vs. placebo	48 weeks	Weight −24.2% (12 mg) vs. −2.1% (placebo); no plateau at 48 weeks	GI events mostly mild–moderate; reduced with 2 mg start; dose-dependent heart-rate increase peaking ~24 weeks
Phase 2 T2D [23]	T2D; n = 281; placebo- and dulaglutide-controlled	36 weeks	HbA1c −2.02 percentage points at 24 weeks (12 mg); weight −16.9% at 36 weeks	GI events; no severe hypoglycemia
Phase 2a MASLD substudy [22]	Obesity with liver fat ≥ 10%; n = 98 (MRI-PDFF)	48 weeks	Relative liver-fat −81.4% (8 mg) and −82.4% (12 mg) at 24 weeks; 79% and 86% reached <5% liver fat	Consistent with parent trial

GI, gastrointestinal; MAD, multiple ascending dose; MRI-PDFF, magnetic resonance imaging proton density fat fraction; PK, pharmacokinetics; SAD, single ascending dose; T2D, type 2 diabetes; t½, elimination half-life; ↓ indicates a decrease; ↑ indicates an increase.

**Table 4:** Handling of intercurrent events and missing data under the two estimands used in the phase 3 trials, as described in the cited reports.

Trial	Efficacy Estimand: Handling of Intercurrent Events	Treatment-Regimen Estimand	Missing-Data Handling Reported
TRIUMPH-1, TRIUMPH-2, TRIUMPH-3 [11,14] (sponsor topline)	Effect had all randomized participants remained on study intervention, with dose interruptions and modifications permitted, and without initiating prohibited weight-management treatments	Average effect regardless of adherence to study intervention or initiation of prohibited weight-management treatments	Not specified in the topline disclosures
TRIUMPH-4 [10] (sponsor topline)	As above for weight and knee pain	Average effect regardless of adherence or initiation of prohibited weight-management treatments; for knee pain, no treatment benefit is assumed if significant actions associated with lack of efficacy were required	Not specified in the topline disclosure
TRANSCEND-T2D-1 [13] (peer-reviewed)	Effect had all randomized participants remained on study intervention for 40 weeks without initiating additional antihyperglycemic medication (more than 14 days of use)	Average effect regardless of adherence or initiation of additional antihyperglycemic medication	Mixed model for repeated measures for the efficacy estimand; multiple imputation under a missing-at-random assumption for the treatment-regimen estimand

Definitions are taken from the cited sponsor disclosures and the peer-reviewed TRANSCEND-T2D-1 report [10,11,13,14]. The efficacy estimand targets a hypothetical outcome under continued treatment and without specified additional or prohibited therapies, whereas the treatment-regimen estimand targets outcomes regardless of these intercurrent events. Neither estimand eliminates bias from incomplete follow-up; estimation depends on the observed data, missing-data assumptions, and sensitivity analyses. Because the TRIUMPH topline disclosures do not state how missing values were imputed, the extent to which the reported differences between the two estimands reflect discontinuation rather than the imputation method cannot be determined from the available reports.

**Table 5:** Adverse events of interest and discontinuation rates in phase 3 trials. All values in this table are taken from sponsor topline disclosures and are not yet supported by full peer-reviewed reports.

Event (% of Participants)	TRIUMPH-1 4/9/12 mg vs. PBO	TRIUMPH-2 4/9/12 mg vs. PBO	TRIUMPH-3 9/12 mg vs. PBO	TRIUMPH-4 9/12 mg vs. PBO
Nausea	28.6/38.4/42.4 vs. 14.8	13.7/20.8/28.0 vs. 8.0	21.7/22.4 vs. 5.8	38.1/43.2 vs. 10.7
Diarrhea	25.2/34.1/32.0 vs. 13.5	27.4/33.5/33.6 vs. 13.2	30.1/24.4 vs. 8.7	34.7/33.1 vs. 13.4
Vomiting	10.6/22.8/25.3 vs. 4.8	5.5/10.2/15.7 vs. 4.2	NR	20.4/20.9 vs. 0.0
Constipation	23.8/25.9/26.1 vs. 10.9	14.0/16.2/16.8 vs. 9.4	18.0/15.7 vs. 7.1	21.8/25.0 vs. 8.7
Dysesthesia	5.1/12.3/12.5 vs. 0.9	4.5/5.6/7.3 vs. 0.7	6.4/6.4 vs. 1.3	8.8/20.9 vs. 0.7
Urinary tract infection	7.5/8.8/8.4 vs. 5.3	3.8/6.3/8.0 vs. 6.6	6.1/7.0 vs. 5.3	NR
Discontinuation due to AEs	4.1/6.9/11.3 vs. 4.9	3.8/11.6/7.7 vs. 4.9	9.8/13.5 vs. 4.8	12.2/18.2 vs. 4.0

Data from refs. [10,11,14], all of which are sponsor topline disclosures rather than peer-reviewed trial reports; analysis populations, denominators, and severity gradings are not fully specified in these sources. For comparison, the peer-reviewed TRANSCEND-T2D-1 report gives adverse-event discontinuation rates of 2.2%, 4.5% and 5.1% with retatrutide 4, 9 and 12 mg versus 0% with placebo [13]. AE, adverse event; NR, not reported in topline disclosure; PBO, placebo. TRIUMPH-4 gastrointestinal frequencies are from the 11 December 2025 sponsor disclosure.

**Table 6:** Pharmaceutical development considerations and open questions for retatrutide.

Domain	Evidence or Premise	Development Questions
Drug substance	Known modified peptide structure; related tirzepatide SPPS/LPPS manufacturing precedent	Retatrutide route, yield, impurity clearance, and scalability require product-specific evidence.
Identity and potency	Three-receptor agonism is intrinsic to the molecule	Connect sequence/acylation identity with validated functional activity at each receptor.
Purity and particles	Structure-informed impurity and aggregation risks	Define CQAs; characterize relevant impurities, qualification needs and formulation-dependent particle formation.
Formulation	Lipidated, amphiphilic peptide	Demonstrate stability, compatibility, container integrity and in-use performance.
Device and delivery	Several clinical escalation doses	Validate dose accuracy, usability, delivery volume and device–formulation compatibility.
Pharmacokinetics	Approximately 6-day half-life in early studies	Assess population variability, organ impairment and exposure–response.
Immunogenicity	Modified peptide acting at three receptors	Evaluate binding and neutralizing ADAs and their clinical relevance.
Regulatory	Sponsor-stated BLA plan for Q1 2027	Regulatory acceptance, classification and final labelling are distinct decisions.
Unapproved supply	FDA warnings about unapproved retatrutide	Characterize identity, content, purity, sterility and endotoxin; document provenance in safety reports.

ADA, anti-drug antibody; BLA, Biologics License Application; CQA, critical quality attribute; FDA, US Food and Drug Administration; LPPS, liquid-phase peptide synthesis; Q1, first quarter; SPPS, solid-phase peptide synthesis.

**Table 7:** Selected obesity-drug trial benchmarks, shown for context rather than head-to-head comparison.

Agent/Selected Dose	Mechanism/Route	Duration	Trial	Weight Change/Estimand
Semaglutide 2.4 mg	GLP-1R; s.c. weekly	68 wk	STEP 1 [4]	−14.9% vs. −2.4% (treatment policy)
Semaglutide 7.2 mg	GLP-1R; s.c. weekly	72 wk	STEP UP [43]	−18.7% vs. −3.9% (treatment policy)
Tirzepatide 15 mg	GIPR/GLP-1R; s.c. weekly	72 wk	SURMOUNT-1 [5]	−20.9% vs. −3.1% (treatment regimen)
Cagrilintide/semaglutide 2.4/2.4 mg	Amylin/calcitonin receptor + GLP-1R; s.c. weekly	68 wk	REDEFINE 1 [44]	−20.4% vs. −3.0% (treatment policy)
Orforglipron 36 mg	GLP-1R (non-peptide); oral daily	72 wk	ATTAIN-1 [45]	−11.2% vs. −2.1% (treatment regimen)
Survodutide 4.8 mg	GLP-1R/GcgR; s.c. weekly	46 wk	Phase 2 [46]	−14.9% vs. −2.8% (planned treatment)
Survodutide 6.0 mg	GLP-1R/GcgR; s.c. weekly	48 wk	SYNCHRONIZE-MASLD [48]	−8.7% vs. −1.4% (treatment regimen); at-risk MASLD population
Retatrutide 12 mg	GIPR/GLP-1R/GcgR; s.c. weekly	80 wk	TRIUMPH-1 [11] (sponsor topline)	−25.0% vs. −3.9% (treatment regimen); −28.3% vs. −2.2% (efficacy)

Values are within-group percentage changes from baseline. The retatrutide values are from a sponsor topline disclosure, whereas the other rows are from peer-reviewed reports. Cross-trial comparisons are limited by population, baseline BMI, duration, background care, missing-data methods, and estimand definitions. The selected trial doses are not a complete or current prescribing guide. The survodutide phase 2 value refers to planned treatment, avoiding comparison with a dose-achieved analysis. Because the estimands, escalation schedules and missing-data methods differ across these trials, the values in adjacent rows are not directly comparable and must not be read as a ranking of agents. GcgR, glucagon receptor; GIPR, glucose-dependent insulinotropic polypeptide receptor; GLP-1R, glucagon-like peptide-1 receptor; MASLD, metabolic dysfunction-associated steatotic liver disease; s.c., subcutaneous; wk, weeks.

## Data Availability

No new data were created or analyzed in this study. Data sharing is not applicable to this article.

## Abbreviations

The following abbreviations are used in this manuscript:

aa	amino acids
ADA	anti-drug antibody
AE	adverse event
AEEA	8-amino-3,6-dioxaoctanoic acid
AHI	apnea–hypopnea index
Aib	α-aminoisobutyric acid
AUC_0–∞_	area under the concentration–time curve from time zero to infinity
BLA	Biologics License Application
BMI	body mass index
cAMP	cyclic adenosine monophosphate
CI	confidence interval
C_max_	maximum plasma concentration
CMC	chemistry, manufacturing and controls
CQA	critical quality attribute
CVD	cardiovascular disease
CYP	cytochrome P450
DPP-4	dipeptidyl peptidase-4
DXA	dual-energy X-ray absorptiometry
EC_50_	half-maximal effective concentration
FDA	US Food and Drug Administration
GcgR	glucagon receptor
GI	gastrointestinal
GIP	glucose-dependent insulinotropic polypeptide
GIPR	glucose-dependent insulinotropic polypeptide receptor
GLP-1	glucagon-like peptide-1
GLP-1R	glucagon-like peptide-1 receptor
γGlu	γ-glutamyl
GMP	good manufacturing practice
HbA1c	glycated hemoglobin
HEK-293	human embryonic kidney 293 (cell line)
HPLC	high-performance liquid chromatography
ICH	International Council for Harmonization
LPPS	liquid-phase peptide synthesis
MACE	major adverse cardiovascular events
MAD	multiple ascending dose
MASH	metabolic dysfunction-associated steatohepatitis
MASLD	metabolic dysfunction-associated steatotic liver disease
α-MeLeu	α-methyl-L-leucine
MRI	magnetic resonance imaging
MRI-PDFF	MRI proton density fat fraction
NR	not reported
NuSH	nutrient-stimulated hormone
OA	osteoarthritis
OSA	obstructive sleep apnea
PBO	placebo
PK	pharmacokinetics
pp	percentage points
Q1	first quarter
SAD	single ascending dose
s.c.	subcutaneous
SEM	standard error of the mean
SPPS	solid-phase peptide synthesis
T2D	type 2 diabetes
t½	elimination half-life
t_max_	time to maximum concentration
wk	weeks
WOMAC	Western Ontario and McMaster Universities Osteoarthritis Index
